# Characterizing lignins from various sources and treatment processes after optimized sample preparation techniques and analysis via ESI-HRMS and custom mass defect software tools

**DOI:** 10.1007/s00216-023-04942-x

**Published:** 2023-09-16

**Authors:** Dane R. Letourneau, Bryan P. Marzullo, Anastasia Alexandridou, Mark P. Barrow, Peter B. O’Connor, Dietrich A. Volmer

**Affiliations:** 1 Department of Chemistry, Humboldt University Berlin, 12489 Berlin, Germany; 2 Department of Chemistry, University of Warwick, Coventry, CV4 7AL UK

**Keywords:** Analytical methods, Lignin, Mass defect, Mass spectrometry, Sample preparation, Custom software

## Abstract

Sample preparation of complex, natural mixtures such as lignin prior to mass spectrometry analysis, however minimal, is a critical step in ensuring accurate and interference-free results. Modern shotgun-MS techniques, where samples are directly injected into a high-resolution mass spectrometer (HRMS) with no prior separation, usually still require basic sample pretreatment such as filtration and appropriate solvents for full dissolution and compatibility with atmospheric pressure ionization interfaces. In this study, sample preparation protocols have been established for a unique sample set consisting of a wide variety of degraded lignin samples from numerous sources and treatment processes. The samples were analyzed via electrospray (ESI)-HRMS in negative and positive ionization modes. The resulting information-rich HRMS datasets were then transformed into the mass defect space with custom R scripts as well as the open-source Constellation software as an effective way to visualize changes between the samples due to the sample preparation and ionization conditions as well as a starting point for comprehensive characterization of these varied sample sets. Optimized conditions for the four investigated lignins are proposed for ESI-HRMS analysis for the first time, giving an excellent starting point for future studies seeking to better characterize and understand these complex mixtures.

**Graphical Abstract:**

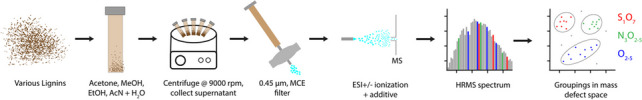

**Supplementary Information:**

The online version contains supplementary material available at 10.1007/s00216-023-04942-x.

## Introduction

Lignin is the most abundant natural aromatic polymer and an exciting renewable resource with a diverse range of applications [1], including fossil fuel replacement in bioethanol production [2] and use in the sustainable production of bulk chemicals [3]. However, its innately complex structure, consisting of a number of common monomers and other substructures [4], which can cross-link in a variety of ways, presents a considerable analytical challenge [5]. In the last few decades, developments in mass spectrometry have vastly expanded the capabilities of the technique for characterizing complex samples (such as NOM [6], crude oil [7], or lignin [8]) without the need for extensive sample preparation or chromatography. However, there are still many factors to consider before a complex natural sample can be introduced to an MS instrument, even when minimal sample preparation is performed. When attempting to retain as many components of the sample as possible in an intact, non-fragmented state, including very high MW components, there needs to be a careful consideration of which solvents, additives, and filtration techniques are employed in the preparation stages.

Lignin can be categorized in various ways — by the source of the lignin, the industrial process from which particular types of lignin are derived, and the pretreatments applied to the lignin for various purposes [9]. Sources of lignin include hardwoods, softwoods, and grasses, which have often undergone industrial pulping processes — including alkaline (Kraft and soda) or organosolv [10] — which can affect the structure and composition of the lignin. Pretreatments can also play a significant role in determining the character of the lignin being studied, including high-pressure steaming (with or without explosion), acid hydrolysis, milling, microwave irradiation, enzymatic and chemical degradation, or biological pretreatment [11]. Many of these pretreatments can induce depolymerization/fragmentation of the lignin. Lignin can also be broadly categorized as sulfur or sulfur-free. Examples of sulfur lignins are lignosulfonates or lignins from the Kraft process [12]. Sulfur-free lignins include soda, alkaline, or organosolv lignins [13]. The source and characteristics of each particular sample can mean that there may be a different ideal combination of sample preparation steps and/or different ionization techniques prior to MS analysis [14]. A small selection of the literature on sample preparation for API-MS of lignin will be presented in this introduction; however, a more comprehensive review is available in the Supporting Information in Table S1.

While some direct injection techniques such as pyrolysis combined with API-HRMS [15] do not require a prior solvent extraction, many others do, and selecting a solvent for dissolution of a polymer can be challenging. Unlike non-polymeric materials, polymers do not dissolve instantaneously and the dissolution is controlled by either the disentanglement of the polymer chains or by the diffusion of the chains through a boundary layer adjacent to the polymer–solvent interface [16]. Lignin is no exception. Although lignin is known to exhibit high solubility in pyridine and DMSO [17, 18], a variety of industrial solvents [19], and various ionic liquids [20, 21], these solvents are not amenable to atmospheric pressure ionization (API) for liquid chromatography-mass spectrometry (LC–MS) and can also be quite harsh, resulting in unwanted reactions or corrosion [14]. In addition, lignins from various sources may be better suited to certain solvent mixtures — for example, organosolv lignin can be dissolved in various organic solvents, with the highest solubility observed in methanol and dioxane [22], whereas LignoBoost and Kraft lignins are better matched with ethanol, acetic acid, or methanol [23]. In general, pure organic solvents (acetonitrile, THF) or water is not usually able to completely dissolve lignin to give a true solution [24]. Molecular dynamics solvation simulations have showed that different water/co-solvent mixtures (e.g., water/acetone, dioxane/water, or THF/water) that exhibit an intermediate polarity can be ideal for the lignin polymer [25], with the optimal ratio depending on the lignin source. Table 1 highlights several solvents used for API-MS analysis of various lignins.

**Table 1:** Solvents used for API-MS analysis of lignins

Lignin type/source	Solvent(s)
Lignosulfonate	Water-soluble [12]
Kraft, LignoBoost	Ethanol, acetic acid, methanol [23]
Organosolv	Organic solvents (MeOH, dioxane) [22]
Steam-exploded corn stalk	4:1 (v/v) ethanol/water [26]
Enzymatic hydrolysis	50% (v/v) ethanol/water [27]
Wheat straw soda	Water (40%):acetone (60%) [28]
Wheat straw organosolv	Water (20–30%):acetone(70–80%) [28]
Softwood Kraft	Water (10–20%):acetone(80–90%) [28]

After dissolving lignin in a suitable solvent, it is still often necessary to perform a filtration step prior to MS analysis to ensure any particulate impurities and/or bacteria [29] are removed. Microfiltration (0.1–0.45 μm) is usually performed for this purpose, although filters of smaller pore sizes can also be used as a way to fractionate lignin into different molecular weights [30]. The membrane material for filtration is an important consideration. Cellulose offers low protein binding characteristics [31] and has been used in filtering “dirty” water samples [32]; however, others have used polytetrafluoroethylene (PTFE) [33, 34] and polyethersulfone (PES) [35, 36] filters for the same reason. Membrane sizes are usually 0.2–0.45 μm.

High-resolution mass spectrometry has long been established as a powerful tool for characterizing lignin [8]. A great variety of MS-based techniques have been utilized for this purpose, including hybrid methods involving chromatography as well as “shotgun” techniques, where a complex mixture is directly injected, often via an API interface, without prior separation. Other studies [14, 37] have extensively compared different API methods for effective ionization of lignin, either degraded or intact. Briefly, APPI has been successfully used to characterize a variety of lignins [38–42], with some noting improved signal intensities and a lower sensitivity to contaminants when compared to APCI and ESI [37]. APCI has been noted for its abilities to more successfully ionize weakly polar lignin molecules, and reduce matrix effects [37]. However, others have observed that APCI and APPI are often limited to a low MW range (< 1800 Da) and singly charged ions, and APCI can result in unwanted fragmentation of lignin in the ion source [24]. Therefore, out of the available API techniques, ESI tends to be the most common choice for lignin analysis. One study found that out of all API techniques surveyed, ESI performed the best for lignin-like species (O/C 0.2–0.6 and H/C 0.7–1.5), and ionized some sulfur-containing lignin species not observed in APCI or APPI [43]. The same study observed that a greater number of elemental formulae were found when using ESI − mode when compared to ESI + or APCI/APPI in either mode [43]. ESI does come with limitations, and given that it performs best in the analysis of polar compounds, it will be best suited for studying high- and medium-polarity lignin components [44]. However, the ubiquity of this technique in the literature makes it easy to compare results to other published research, and it is therefore an obvious choice when selecting a single ionization technique to compare the various sample preparation conditions that are the focus of this study.

Once HRMS spectra have been collected, there are numerous techniques to untangle and interpret the highly complex data [39, 45, 46]. Among the most popular are Kendrick mass defect plots, which exploit high-accuracy mass information to transform MS data into the so-called mass defect space, revealing patterns due to repeating structural motifs [47]. Several recent studies have employed this technique to remove undesired mass spectral features [48], process congested spectra of polymers with multiple charges [49], and screen for poly- and perfluroalkyl substances in contaminated soil [50], among many other studies including numerous analyses of lignin [39]. Custom algorithms and software tools have also played a major role in many recent publications when processing the data resulting from HRMS analyses of complex samples such as lignin [51–56]. Among these are various open-source software solutions, including the web-based software Constellation (previously developed in-house), which allows expansion and manipulation of HRMS data into the mass defect space, as well as algorithms which are able to automatically find repeating patterns (potentially corresponding to repeating units in polymers or molecules with moieties of changing mass) or assign molecular formulae to masses in the HRMS dataset [57, 58].

In this work, we present the optimized sample preparation of a unique sample set of lignins from various sources and treatment processes, followed by characterization via ESI-HRMS and subsequent data analysis with custom mass defect analysis software tools developed in-house. Although a number of reviews have been conducted of various mass spectrometry-based analytical techniques for lignin (MALDI-MS [59], API-MS [37], ESI–MS [24]), to our knowledge, this is the first study attempting to systematically optimize conditions for ESI-HRMS analysis of a highly varied set of lignin samples. After deciding on sample preparation protocols and selecting a set of solvent mixtures to test, we performed HRMS experiments and used custom mass defect analysis software to transform and visualize the spectra in the mass defect space, as well as to assign molecular formulae based on the exact masses. From this analysis, we were able to characterize several groupings visible in the mass defect space based on their mass and mass defect ranges, as well as DBE, H/C and O/C ratios, and any heteroatom classes present. Of particular interest are the noted differences in these groupings between lignin samples from various sources and pretreatments, and between the different solvents used for extraction and ionization. In general, we observed that the choice of solvent mixture is of critical importance when optimizing sample preparation for degraded versus non-degraded lignins, and for lignins from different sources. We found that depending on the lignin source or treatment, use of a particular solvent mixture versus another may result in some components of the lignin (visible as groupings of peaks in the mass defect space) not being fully extracted or ionized in an API source. Solvent choice and preparation conditions are therefore important considerations before ionization and HRMS analysis when attempting to acquire a complete picture of any given lignin sample being studied.

## Materials and methods

### Chemicals and materials

HPLC–MS grade acetonitrile (AcN) was obtained from Chemsolute (Th. Geyer GmbH & Co. KG, Renningen, Germany); methanol (MeOH) from VWR Chemicals (Darmstadt, Germany); and acetone (AC) and ethanol (EtOH) from Carl Roth GmbH & Co. KG (Karlsruhe, Germany). Organic-free water (H_2_O) was generated by a Millipore (Bedford, MA, USA) Direct-Q8 purification system.

### Lignin samples

A large variety of powdered lignin samples from various sources and process types were kindly provided by LignoPure GmbH [60]. From these samples, we chose four (LP3, LP5, LP7, LP9) to represent lignin originating from different sources (i.e., hardwood, softwood), having undergone various treatments (i.e., organosolv, steam treatment) and in different states of degradation (i.e., having undergone enzymatic hydrolysis or not). Table 2 lists the investigated samples along with their associated process type and Klason lignin content.

**Table 2:** Studied lignin samples

Code	Sample (process type)	Lignin descriptor	Klason lignin
LP3	Beech wood lignin (organosolv, EtOH + H_2_O)	Hardwood, no degradation	> 95%
LP5	Miscanthus lignin (steam treatment + EH)	Grasses, degraded	70–80%
LP7	Spruce lignin (dilute acid + EH)	Softwood, degraded	75%
LP9	Hardwood lignin (2G biorefinery, pretreatment + EH)	Hardwood, degraded	84–88%

### Solvent mixtures

Ten different solvent mixtures were evaluated in terms of dissolution for lignin and minimum background noise and enhanced ionization during MS analysis: acetone:H_2_O (1:1, v/v), MeOH:H_2_O (1:1, v/v), EtOH:H_2_O (1:1, v/v), AcN:H_2_O (1:1, v/v), MeOH:H_2_O (3:1, v/v), AcN:H_2_O (3:1, v/v), MeOH:H_2_O (1:3, v/v), AcN:H_2_O (1:3, v/v), acetone:AcN:H_2_O (1:1:2, v/v/v), acetone:MeOH:H_2_O (1:1:2, v/v/v). The selection of these solvents was based on an extensive review of the literature regarding known preparations of lignin and lignin model compounds prior to direct analysis by API-MS, available in Table S1 in the Supporting Information. The solvent mixtures along with their associated organic/aqueous ratios are listed in Table 3.

**Table 3:** Solvent mixtures used in sample preparation

Solvent mixture	Organic/aqueous ratio
Acetone:H_2_O (1:1)	0.5
MeOH:H_2_O (1:1)	0.5
EtOH:H_2_O (1:1)	0.5
AcN:H_2_O (1:1)	0.5
MeOH:H_2_O (3:1)	0.75
AcN:H_2_O (3:1)	0.75
MeOH:H_2_O (1:3)	0.25
AcN:H_2_O (1:3)	0.25
Acetone:AcN:H_2_O (1:1:2)	0.5
Acetone:MeOH:H_2_O (1:1:2)	0.5

### Sample preparation of lignin

Ten milligrams of each lignin sample was dissolved in 4 mL of each solvent mixture at room temperature and then vortexed for 1 min. Subsequently, centrifugation at 9000 rpm took place for 7 min, after which 0.2 mL of the supernatant was transferred to 10 mL of the same solvent mixture. Finally, the diluted sample was filtered through a Millex syringe filter (0.45 µm pore size, 25 mm diameter, Millex-HA mixed cellulose esters membrane, hydrophilic, Merck Millipore Ltd., Tullagreen, Carrigtwohill, Co Cork, Ireland) prior to storage in the final sample container to ensure that there were no remaining particles. In addition, solvent blanks were included in this sample preparation process, undergoing the exact same treatment as the lignin samples. The resulting sample blank spectra were subsequently subtracted from the spectra of the lignin-containing samples to try and mitigate any potential contamination from polar components at this stage.

It should be noted that although all samples underwent a full solvent extraction, as is standard for most studies in the literature analyzing lignin via API-MS, not every sample fully dissolved in each solvent mixture, and it is therefore possible that there were a variety of lignin components not extracted during sample preparation due to their high MW and low solubility. This would render these lignin components invisible in our analyses, and this is considered when discussing results and comparing samples.

### Mass spectrometry

Prior to MS analysis, samples were diluted tenfold in their respective solvents. To some samples, 0.1% formic acid was added to the diluted solutions to enhance protonation. All samples including solvent blanks were analyzed on a 15-T Bruker Solarix XR FT-ICR (Bruker, Bremen, Germany) using a dynamically harmonized cell in 1-omega mode acquired with a 4-megaword (32-bit integer) transient. Two hundred average scans were acquired for all data files with an accumulation time of 0.2 s in broadband mode. No phasing was performed on the data. Data was acquired in both positive and negative ion modes. Samples were ionized using a home-built nESI source with a nichrome wire placed into the sample for the ground electrical connection. All samples were directly infused into the mass spectrometer.

All data files (samples and solvent blanks) were calibrated externally using Hpmix (Agilent Technologies, Santa Clara, USA), selecting reference peaks that covered the entire *m/z* range of the samples. Once calibrated, solvent blanks were subtracted from the samples to minimize peaks not related to the sample itself. The solvent subtracted data files were then exported as “.xy” (ASCII text files) for analysis, summarized as two columns of data, “*m/z*” (mass-to-charge ratio) and “I” (intensity). Data calibration, subtraction, and extraction were performed in Bruker DataAnalysis 5.0 software.

### Data processing

All data files were loaded into RStudio 2022.07.2 Build 576 via custom R scripts, where data processing and analysis were then carried out with the help of various R packages including tidyverse, dplyr, and gatepoints [61] as well as default built-in R functions. All peaks with less than 1% normalized intensity were filtered out of all mass spectra unless otherwise noted. Figures were first generated using ggplot2 [62] in R and then exported as “.png” files and in some cases, further customized in Adobe Illustrator 2022.

Molecular formula finding was performed using the open-source CoreMS *SearchMolecularFormulas* function [63] built into the Constellation mass defect analysis web application [57] previously developed by our group. This application allows users to upload their raw MS data, transform it into the mass defect space, and perform various data analysis and visualization functions, including molecular formula finding. Settings for molecular formula finding are fully user-selectable, and for this study, the parameters were as follows: minimum error − 0.5 ppm, maximum error 0.5 ppm; minimum DBE 0, maximum DBE 50; MS noise threshold 3; elemental limits: C 1–90, H 4–200, O 1–23, N 0–5, S 0–1. All other settings were left to their defaults in Constellation. These defaults include a search for all isotopes including automatic fine isotopic structure calculation [63], and exclusion of adducts from elemental composition determination. In the case that multiple molecular formulae matched a particular HRMS peak, the formula with the lowest *m/z* error (highest mass accuracy) was chosen. However, as with any complex mixture, the assignment of molecular formulae using only high-resolution mass information is decidedly tentative, and caution should be used in interpreting results. The resulting “.csv” files of formula information (including molecular formulae, heteroatom classes, DBE, and H/C and O/C ratios) were then loaded into R, and the information was matched with corresponding peaks in the HRMS spectra via the index of each point from the raw MS data files.

## Results and discussion

A diverse set of lignin samples were prepared in various solvents, ionized via ESI in both positive and negative modes, and analyzed via HRMS and custom mass defect analysis software. The results from all stages of the study indicated a high level of variation in spectral composition among both samples from various sources and treatment processes, and different solvent mixtures used in extraction and ionization. A summary of the experiments conducted is presented in Table 4, which are described in detail individually in the following sections.

**Table 4:** Summary of experiments conducted

Sample(s)	Experiment	Goal
Non-degraded hardwood lignin LP3	Compare all solvent mixtures under 3 different ionization conditions	Identify the solvent mixture able to extract/ionize the largest number of lignin components in each ionization mode
Non-degraded hardwood lignin LP3	Compare all solvent mixtures in ESI − mode	Identify solvent mixtures which extract/ionize the largest number of lignin components for all peak groupings
Non-degraded hardwood lignin LP3	Compare all solvent mixtures in ESI + mode	Identify solvent mixtures which extract/ionize the largest number of lignin components for all peak groupings
2 hardwood lignins, one degraded (LP9) and one non-degraded (LP3)	Compare degraded versus non-degraded lignins	Characterize the differences in peak groupings and optimized solvent mixtures for degraded versus non-degraded lignins
3 degraded lignins from softwood (LP7), hardwood (LP9), and grasses (LP5)	Compare lignins from softwood, hardwood, and grasses	Characterize the differences in peak groupings and optimized solvent mixtures for lignins from softwood, hardwood, and grasses

### General qualitative observations during sample preparation

During sample preparation, qualitative observations were noted regarding how each powdered lignin sample interacted with each solvent mixture upon contact and mixing (after vortexing, but prior to the centrifugation and filtering steps). A “variation score” from 1–3 was established, where samples were ranked according to how much variation they exhibited after their initial mixing with the 8 different solvent mixtures: 1 = very little variation, 2 = some variation, 3 = dramatic variation between solvent mixtures. Variation was observed for 3 categories: color, residue, and “milkiness.” We then summed these variation scores to give a total score out of 9, and then ranked the samples according to this total score, in order to select a lignin exhibiting high variation among solvents for an initial set of HRMS experiments. The full results are summarized in the Supporting Information (Table S2).

The hardwood lignin LP3 from the organosolv process, with no prior enzymatic degradation, exhibited the highest qualitative variation score (8) during this sample preparation stage and was thus chosen for an initial run of experiments comparing all solvent mixtures under 3 different ionization conditions, with the expectation that these qualitative observations of variation would translate into distinct spectral differences after HRMS and data analysis.

### Characterization of a non-degraded hardwood lignin sample

**Fig. 1:**
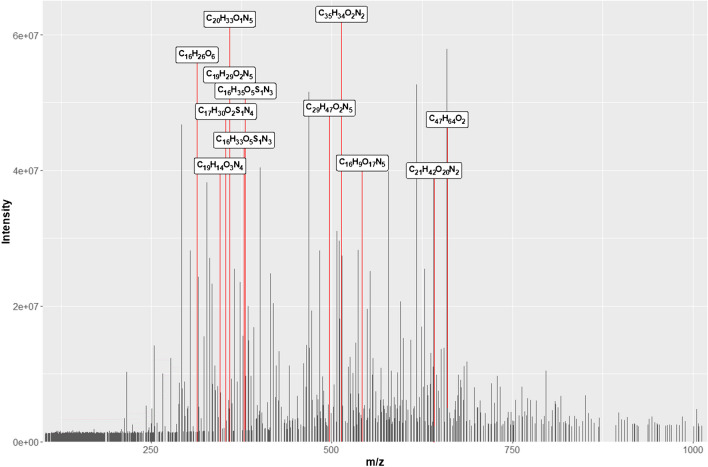
ESI( −)-HRMS spectrum of a hardwood, non-degraded lignin (LP3) in 1:1 AcN:H_2_O, partially annotated with molecular formulae from the CoreMS molecular formula finding algorithm

Full-scan mass spectra were measured from *m/z* 100–2000 for the LP3 sample in all solvent mixtures in both ESI − and ESI + modes (ESI + mode, with and without formic acid). From each spectrum, we subtracted the solvent blank spectrum, and exported a mass list to a text file. We then calculated molecular formula information for each peak using the molecular formula finding algorithm from CoreMS [63], running in the Constellation software environment [57], as described in the “Materials and methods” section. The resulting spectra were highly complex, containing hundreds to thousands of peaks, many with an associated molecular formula. Figure 1 shows an example of one of these spectra for the LP3 sample in 1:1 AcN:H_2_O, ionized in ESI − mode, and partially annotated (for clarity’s sake) with molecular formula information. As in the analysis of many complex mixtures via HRMS, these are tentative molecular formulae assignments, and should be interpreted with caution.

The total number of peaks in each spectrum (corresponding to the total number of non-background ions observed) was used as an initial step to ascertain how much ionizable lignin material each solvent mixture had extracted during sample preparation. For each sample set (ESI − , ESI + , ESI + with formic acid), the data was sorted according to the number of peaks observed in each spectrum. The full results are summarized in the Supporting Information (Table S3).

Results showed that for this hardwood, non-degraded lignin, the best solvent mixture for both positive and negative ionization modes was 1:1 AcN:H_2_O, and the best for positive mode with formic acid addition was 1:1 MeOH:H_2_O. On average, there were slightly more ions observed in ESI + mode without formic acid added than with (~ 10% increase), and a wealth of additional ions observed in negative over positive ion mode (~ 200% increase). Overall rankings suggest that solvents with an organic/aqueous ratio of 0.5 or greater are ideal for this lignin sample and that 1:1 AcN:H_2_O is likely the best choice over all ionization modes. However, this only represents one strategy for ranking the effectiveness of these solvent mixtures and does not consider more subtle variances in spectrum composition which may be worth considering and are therefore discussed next.

### Characterization in ESI − mode across all solvent mixtures

**Fig. 2:**
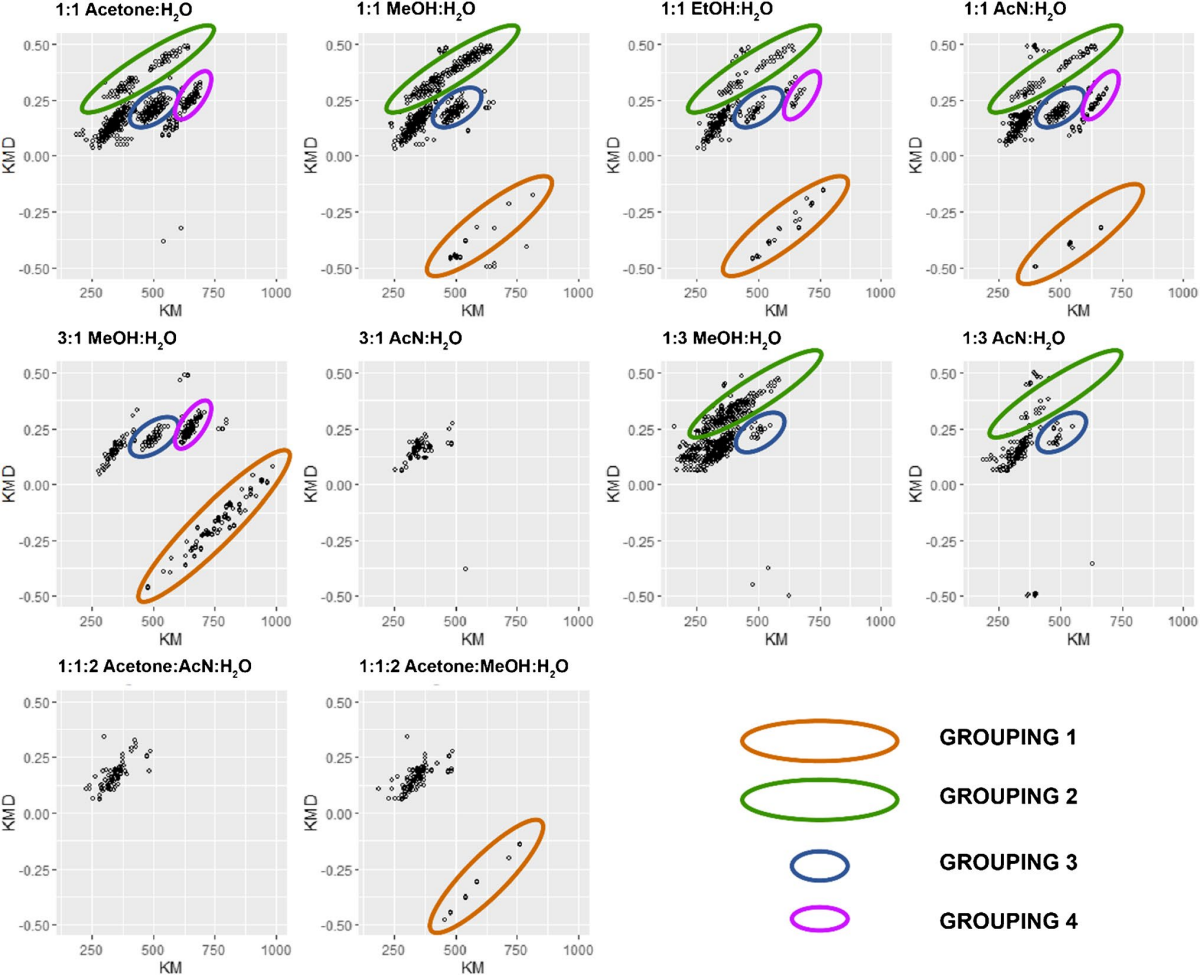
LP3, a non-degraded hardwood lignin, extracted and ionized with all solvent mixtures in ESI − mode, analyzed via HRMS with peaks < 1% intensity filtered out, and transformed into the mass defect space with base CH_2_ (14/14.01565). Four groupings of peaks are identified and circled in each graph

As previously observed [39, 45, 49, 57, 64], transforming a high-resolution mass spectrum into the mass defect space can allow for the insightful observation of patterns and/or groupings of peaks in the spectra. Here, we wanted to use this transformation to better characterize the effectiveness of each solvent mixture used in sample preparation in extracting and ionizing various components of our lignin mixtures. The same set of spectra collected for the hardwood, non-degraded lignin (LP3) in the previous step were transformed into the mass defect space via the CH_2_ base (nominal mass 14, exact mass 14.01565). In the resulting graphs of Kendrick mass versus Kendrick mass defect, we observed four distinct groupings of peaks, as shown in Fig. 2. An area with < 5 peaks was not considered to be a group. Each grouping was then selected directly on the graphs (generated using ggplot2 [62] in R) using the gatepoints [61] package. This gave a list of indices for each point in the grouping, allowing us to match each point with any corresponding molecular formula information.

As previously mentioned, for each spectrum, we ran the molecular formula finding algorithm from CoreMS [63], running on a server at HU Berlin and accessible via an interface built into the Constellation mass defect analysis software developed by our group [58]. This molecular formula data allowed us to better characterize these groupings of peaks and make distinctions not only based on their positioning in the mass defect space (KM and KMD), but also based on their DBE, H/C and O/C ratios, and heteroatom classes present. These data are summarized in Table 5.

**Table 5:** Summary of groupings observed for LP3 in ESI − mode

Group	Optimum 3 solvents	KM	KMD	H/C	O/C	DBE	Unique heteroatom classes
1	3:1 MeOH:H_2_O 1:1 EtOH:H_2_O 1:1 MeOH:H_2_O	478 to 1081	− 0.47 to 0.22	0.3 to 2.1	0.01 to 1.2	0 to 49	S_0-1_N_1-5_O_>20_
2	1:1 MeOH:H_2_O 1:3 MeOH:H_2_O 1:1 AcN:H_2_O	279 to 643	0.2 to 0.5	0.4 to 2.3	0.02 to 1.2	0 to 34	O_4-17_
3	1:1 acetone:H_2_O 1:1 MeOH:H_2_O 3:1 MeOH:H_2_O	433 to 569	0.15 to 0.29	1.1 to 2.1	0.03 to 0.42	0 to 17	N_5_O_2-4_
4	3:1 MeOH:H_2_O 1:1 acetone:H_2_O 1:1 AcN:H_2_O	563 to 707	0.09 to 0.33	1.1 to 2.2	0.02 to 0.44	0 to 22	N_1_O_9-11_, N_3_O_1-10_

Based on these results for the hardwood, non-degraded lignin LP3 in all solvent mixtures in ESI − mode, we can summarize our observations as follows:

- Groupings 3 and 4 contain compounds with higher H/C ratios, lower O/C ratios, and lower DBE than groupings 1 and 2
- Solvent mixtures with an organic/aqueous ratio ≥ 0.5 provide improved extraction and ionization of compounds in groupings 1 and 4
- Solvent mixtures with an organic/aqueous ratio ≤ 0.5 exhibit better extraction and ionization of compounds in groupings 2 and 3
- The solvent mixtures which extracted and ionized the largest number of components for all groups were 1:1 EtOH:H_2_O and 1:1 AcN:H_2_O
- 3:1 AcN:H_2_O and 1:1:2 acetone:AcN:H_2_O did not extract or ionize any material from any of the 4 groupings and were therefore the worst solvent mixture choices for this lignin sample in ESI − mode

### Characterization in ESI + mode across all solvent mixtures

**Fig. 3:**
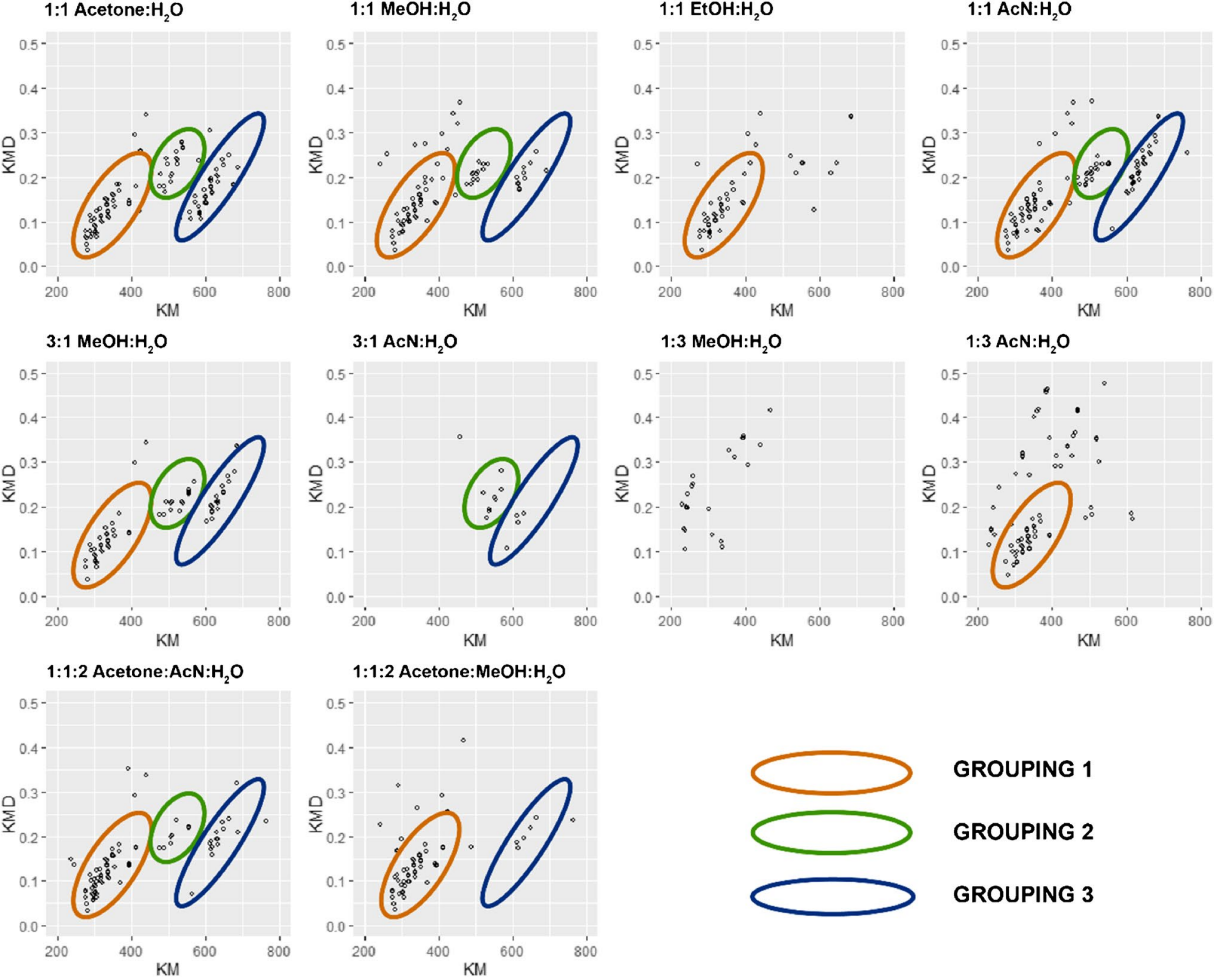
LP3, a non-degraded hardwood lignin, extracted and ionized with all solvent mixtures in ESI + mode, analyzed via HRMS with peaks < 1% intensity filtered out, and transformed into the mass defect space with base CH_2_ (14/14.01565). Three groupings of peaks are identified and circled in each graph

An identical process of identifying and characterizing groups of peaks was conducted for mass defect-transformed HRMS spectra of the same non-degraded hardwood lignin (LP3) for all solvent mixtures in ESI + mode. Figure 3 displays these spectra in the base CH_2_ (14/14.01565) mass defect space, where we observed three distinct groupings of peaks. As before, an area with < 5 peaks was not considered to be a group.

As before, this was followed by further characterization of these groupings via DBE, H/C and O/C ratios, and heteroatom classes obtained from the molecular formula calculations. These data are summarized in Table 6.

**Table 6:** Summary of groupings observed for LP3 in ESI + mode

Group	Optimum 3 solvents	KM	KMD	H/C	O/C	DBE	Unique heteroatom classes
1	1:1 MeOH:H_2_O 1:1 AcN:H_2_O 1:1:2 acetone:AcN:H_2_O	275–356	0.07–0.2	1.4–2.4	0.09–0.31	0–8	N_3_O_2-5_
2	1:1 acetone:H_2_O 1:1 AcN:H_2_O 3:1 MeOH:H_2_O	446–555	0.15–0.24	1.5–2.1	0.03–0.26	0–9	S_1_O_7_
3	1:1 acetone:H_2_O 1:1 AcN:H_2_O 3:1 MeOH:H_2_O	584–688	0.1–0.34	1.5–2.2	0.02–0.28	1–12	O_2-5_, N_2_O_2-4_, N_3_O_7-8_, S_1_N_5_O_5_

Based on these results for the hardwood, non-degraded lignin LP3 in all solvent mixtures in ESI + mode, we can summarize our observations as follows:

- Groupings 2 and 3 share the same optimum solvents and contain compounds with similar H/C and O/C ratios
- Grouping 3 contains compounds with higher DBE than groupings 1 and 2
- Solvent mixtures with an organic/aqueous ratio ≥ 0.5 performed better at extracting and ionizing compounds in groupings 2 and 3
- The solvent mixtures which extracted and ionized the largest number of components for all groups were 1:1 acetone:H_2_O, 1:1 AcN:H_2_O, and 3:1 MeOH:H_2_O
- 1:3 MeOH:H_2_O did not extract or ionize any material from any of the 3 groupings and was therefore the worst choice for this lignin sample in ESI + mode

### Comparison of lignin samples across all solvent mixtures and ionization modes

From our inventory of samples, we selected another three lignins in addition to the hardwood, non-degraded lignin (LP3) to analyze with all of our solvent mixtures in both ionization modes: degraded lignins from grasses (LP5), degraded lignins from softwood (LP7), and degraded lignins from hardwood (LP9). This selection allowed us to compare lignins from three different sources (hardwood, softwood, grasses) and those which have been degraded via enzymatic hydrolysis versus those which have not. An identical data analysis process from the previous sections was conducted for these samples, where groupings of peaks were categorized for each ionization mode and characterized via information gathered from molecular formula data.

### Characterizing degraded versus non-degraded lignins

Comparing two hardwood lignins, one degraded via enzymatic hydrolysis (LP9) and one left intact (LP3), it was clear that there were significant differences in both the amount of extracted/ionized material and the groupings of peaks observed in the mass defect-transformed HRMS spectra. In ESI + mode, the differences in number of peaks and variety of groupings extracted and ionized were significantly impacted by the choice of solvent mixture. More components of the degraded lignin (LP9) were extracted/ionized with a low organic/aqueous ratio (< 0.5) solvent mixture, whereas more components of the non-degraded lignin (LP3) were extracted/ionized with solvents with an organic/aqueous ratio of 0.5. The degraded lignin also exhibited several groupings of peaks (groupings 4 and 5) not observed in the non-degraded lignin. The characteristics of these unique groupings are presented in Table 7.

**Table 7:** Unique groupings observed for LP9 in ESI + mode

Group	Optimum 3 solvents	KM	KMD	H/C	O/C	DBE	Unique heteroatom classes
4	3:1 AcN:H_2_O	819–1111	0.24–0.46	1.3–2.1	0.01–0.4	0–28	O_4-15_, N_2_O_10-18_, N_4_O_6-13_, N_5_O_6-8_, S_1_O_10-15_, S_1_N_3_, S_1_N_5_O_3-13_
5	1:3 AcN:H_2_O 1:1 acetone:H_2_O 1:1 AcN:H_2_O	364–677	0.24–0.5	0.5–2.5	0.02–1.2	0–25	S_1_N_2_O_10-13_

In ESI − mode, differences between solvent mixtures were less pronounced and groupings of peaks looked similar between samples. For both hardwood lignin samples, solvent mixtures with a higher organic/aqueous ratio (0.75, e.g., 3:1 AcN:H_2_O) performed poorly in this mode. The same poor performance was observed for tri-solvent mixtures (e.g., 1:1:2 acetone:MeOH:H_2_O, 1:1:2 acetone:AcN:H_2_O) for both the degraded and non-degraded samples in this mode. Table 8 summarizes the observations and suggests optimized solvent mixtures for each lignin and each ionization mode.

**Table 8:** Optimized solvent mixtures for degraded versus non-degraded lignins

Solvent category	Solvent mixture (organic/aqueous ratio)	
	Hardwood lignin, non-degraded (LP3)	Hardwood lignin, degraded (LP9)
Best for ESI − mode	1:1 EtOH:H_2_O or 1:1 AcN:H_2_O (0.5)	1:3 AcN:H_2_O (0.25)
Worst for ESI − mode	3:1 AcN:H_2_O or 1:1:2 acetone:AcN:H_2_O (0.5–0.75)	3:1 AcN:H_2_O, 1:1 MeOH:H_2_O or 1:1:2 acetone:AcN:H_2_O (0.5–0.75)
Best for ESI + mode	1:1 AcN:H_2_O or 1:1 acetone:H_2_O (0.5)	1:3 AcN:H_2_O (0.25)
Worst for ESI + mode	1:3 MeOH:H_2_O (0.25)	1:1:2 acetone:AcN:H_2_O (0.5)
Best overall	1:1 AcN:H_2_O (0.5)	1:3 AcN:H_2_O (0.25)

### Characterizing lignin from softwood, hardwood, and grasses

We also compared lignins across various sources that had otherwise experienced a similar treatment process (i.e., all had undergone degradation via enzymatic hydrolysis). We chose three degraded lignins from softwood (LP7), hardwood (LP9), and grass (*Miscanthus*, LP5) sources. In ESI + mode, differences in the spectra between the three samples were quite pronounced across the various solvent mixtures. For both the softwood lignin (LP7) and the lignin from grasses (LP5), the solvent mixture of 1:1:2 acetone:AcN:H_2_O extracted a grouping of peaks (grouping 5) not seen to this degree or at all in other solvents or in the hardwood lignin (LP9) in this mode. 1:1:2 acetone:AcN:H_2_O also ranked as the best solvent in positive ion mode for LP5 and 7, whereas it performed the worst for LP9. For all 3 samples in this mode, the solvent mixture of 3:1 AcN:H_2_O was the best at extracting/ionizing a grouping of peaks (grouping 4) common to all samples.

In ESI − mode, differences in spectra were less obvious between solvents and groupings of peaks looked similar between samples. However, the similarities between the softwood and grass lignins (LP5 and LP7) and their shared differences in comparison with the hardwood lignin (LP9), which were observed in ESI + mode, were also observed in ESI − mode. Specifically, the mixtures of 1:1 AcN:H_2_O and 1:1:2 acetone:AcN:H_2_O ranked highly for LP5 and 7, but not for LP9, and the mixture of 1:3 MeOH:H_2_O ranked highly for LP9 but not for LP5 and 7. In general, across both ionization modes, it seems that the hardwood lignin sample (LP9) seemed better suited to solvent mixtures with lower organic/aqueous ratios than the grass and softwood lignins (LP5 and 7). Table 9 summarizes these results.

**Table 9:** Comparing lignins from softwood, hardwood, and grass (*Miscanthus*)

Solvent category	Solvent mixture (organic/aqueous ratio)		
	Softwood lignin, degraded (LP7)	Hardwood lignin, degraded (LP9)	*Miscanthus* lignin, degraded (LP5)
Best for ESI − mode	1:1 AcN:H_2_O 1:1:2 acetone:AcN:H_2_O 3:1 AcN:H_2_O (0.5–0.75)	1:3 AcN:H_2_O (0.25)	1:1:2 acetone:MeOH:H_2_O 1:1 AcN:H_2_O (0.5)
Worst for ESI − mode	1:1 acetone:H_2_O 1:3 MeOH:H_2_O (0.25–0.5)	3:1 AcN:H_2_O 1:1 MeOH:H_2_O or 1:1:2 acetone:AcN:H_2_O (0.5–0.75)	1:1 acetone:H_2_O 1:3 MeOH:H_2_O (0.25–0.5)
Best for ESI + mode	1:1:2 acetone:AcN:H_2_O 3:1 AcN:H_2_O (0.5–0.75)	1:3 AcN:H_2_O (0.25)	1:1:2 acetone:AcN:H_2_O (0.5)
Worst for ESI + mode	1:1 MeOH:H_2_O (0.5)	1:1:2 acetone:AcN:H_2_O (0.5)	1:3 MeOH:H_2_O (0.25)
Best overall	1:1:2 acetone:AcN:H_2_O 3:1 AcN:H_2_O (0.5–0.75)	1:3 AcN:H_2_O (0.25)	None

## Conclusions

In this work, we have demonstrated how the choice of solvent mixture during sample preparation, extraction, and ionization steps can drastically affect the results of ESI-HRMS analyses of powdered lignin samples. We have presented a sample preparation method involving a number of different solvent mixtures for a variety of lignins from various sources and treatment processes, followed by analysis via ESI-HRMS in both positive and negative ionization modes. We then characterized these lignins using different procedures, including assigning tentative molecular formulae and identifying groupings of peaks within the detailed HRMS spectra after transformation into the mass defect space and analysis with custom software routines. Significant differences were noted in these groupings, which varied with solvent mixture, lignin source, and the degree of lignin degradation. Through this, we have suggested optimized solvent choices for sample preparation of various lignins depending on lignin origin and treatment process.

Our results indicate that differences between lignin samples from various sources are much more pronounced in positive ion mode, and lignins from grasses and softwood (LP5 and 7) have more groupings of peaks in common with each other than they do with hardwood lignin (LP9) after enzymatic hydrolysis (degradation). However, if degradation has not occurred for all samples, the differences when comparing between various sources are less pronounced (as would be expected, as here the results are not truly comparable). In general, we observe that the choice of solvent mixture is very important when optimizing sample preparation for degraded versus non-degraded lignins, and for lignins from different sources.

### Supplementary Information

Below is the link to the electronic supplementary material.

Supplementary file1 (PDF 296 KB)
