# Supplementary material for: Characterizing lignins from various sources and treatment processes after optimized sample preparation techniques and analysis via ESI-HRMS and custom mass defect software tools
Source: Anal Bioanal Chem. 2023 Sep 16;415(27):6663–75. doi: 10.1007/s00216-023-04942-x (PMC10598097; doi:10.1007/s00216-023-04942-x)
Supplement: Supplementary file 1 — Supplementary file1 (PDF 296 KB) [file 216_2023_4942_MOESM1_ESM.pdf]

Supporting Information

Sample Preparation Techniques for API-MS of Lignin

Table S1 represents an extensive review of the known preparations of lignin and lignin model compounds prior to direct analysis by API-MS.

Table S1: Sample Preparation Techniques for API-MS of Lignin

Sample	Preparation	API	API solvent	Additive	[Final] lignin	Ref.
Dioxane, Kraft, and organosolv lignins from spruce and eucalyptus	Lignin solutions prepared using MeOH:H_2O (1:1) + 2.5% NH_3 or dioxane:H_2O (7:3)	ESI-	MeOH	0.25% NH_3	0.05% w/v	Evtuguin (1999)[1]
Low MW fraction of *E. globulus* dioxane lignin	Low MW lignin fraction was dissolved in AcN and diluted w/ MeOH	ESI-	MeOH:H_2O (1:1 v/v)	0.25 wt.-% NH_3	0.15 mg/mL	Evtuguin (2003)[2]
Lignin oligomers obtained by thioacidolysis	Oligomers were dissolved directly in the API solvent mix	ESI+	EtOH:H_2O (7:3)	4% CH_3COOH	N/A	Önnerud (2003)[3]
		ESI-	IPA:H_2O (6:4) or EtOH:H_2O (7:3)	4% NaOH		
Wheat straw lignin	Lignin was dissolved in chloroform:MeOH (2:1)	APCI+	$CHCl_3$:MeOH (2:1)	None	N/A	Banoub (2003)[4]
		APCI-				
Wheat straw lignin	~0.1mg of lignin dissolved in either 1 mL dioxane or 1 mL dioxane:MeOH:$CHCl_3$ (1:1:1)	APPI+	MeOH:$CHCl_3$ (1:1)	Toluene	N/A	Banoub (2007)[5]
Synthesized oligolignols	Oligolignols were dissolved in water to 100 μM	APCI-	H_2O:MeOH (50:50 v/v)	0.1% acetate	~3.2 μM	Morreel (2010)[6,7]
Lignin model compounds	Analytes dissolved in 50:50 or 20:80 (v/v) MeOH:H_2O (for analytes with an -OH)	ESI-	MeOH:H_2O (50:50 v/v or 20:80 v/v)	0.1% HCOOH	0.01-1 mg/mL	Amundson (2011)[8]
Steam-exploded wheat straw, bagasse, and organosolv lignins	Lignin (5 mg) was dissolved in 9:1 DMSO/MeOH (10 mL)	ESI+	DMSO/MeO H (9:1)	None	0.5 mg/mL	Dauria (2012)[9]
Lignin model compounds	Stock solutions were prepared at 1 mM in MeOH:H_2O (1:1 v/v)	ESI-	MeOH/H_2O (1:1)	1% NaOH	0.005 mM	Haupert (2012)[10]
		ESI+		5% NaCl		
		APCI-		None		
		APCI+				
Bio-oils, lignin model compounds	Model compounds and bio-oil samples dissolved in MeOH:H_2O (1:1 v/v) at 10 mg/mL	ESI-	MeOH:H_2O (1:1 v/v)	1 mg/mL NaOH, 0.5 mg/mL NH_4Cl	0.01-2 mg/mL	Alsbou (2014) [11]
		ESI+		0.1 mg/mL HCOOH, 0.5 mg/mL NH_4Cl, 0.5 mg/mL NaCl		
Dioxane lignin	Lignin dissolved in acetone:H_2O (9:1)	ESI-	Acetone:H_2O (9:1)	0.1-4% NH_3	50 mg/L	Kosyakov (2016)[12]
		APCI-		None		
		APPI-				
34 lignin model compounds	1 mm solutions of all compounds were prepared in MeOH:H_2O (50:50 v/v)	ESI-	MeOH:H_2O (50:50 v/v)	1% NaOH	N/A	Marcum (2016)[13]
Electrochemical lignin breakdown products	100 μg dissolved in 1 mL of MeOH	ESI-	MeOH	1% CH_3COOH or 1% NH_4OH	100 ng/μL	Dier (2016)[14]
		APCI-				

	100 ng/µL solution in MeOH/Tol (90:10 v/v)	APPI-	MeOH:Tol (90:10 v/v)	None		
Electrochemical lignin breakdown products	Powder was dissolved in H_2O:MeOH:NH_4OH (50:50:1 v/v/v)	ESI-	H_2O:MeOH (1:1 v/v)	1% NH_4OH	N/A	Qi (2016)[15]
Coniferous hydrolysis lignin	Lignin was dissolved in acetone:H_2O (9:1) to 50 ng/µL final conc.	APPI-	Acetone	None	N/A	Kosyakov (2017)[16]
Lignin oligomers w/ β-O-4 and 5-5 linkages	0.1 mM stock solutions prepared in MeOH, then dissolved in AcN to a final vol. of 1 mL and conc. of 0.01 mM	ESI-	AcN	None	0.01 mM	Sheng (2017)[17]
Lignin model compounds (mono-, di-, and trimers), Alkali lignin	Model compounds were dissolved in MeOH:H_2O (1:1) to 100 ppm (w/v). Alkali lignin was dissolved in AcN:H_2O (1:1) or H_2O:THF (1:1) to 100 or 1000 ppm respectively	ESI-<br>ESI+	AcN:H_2O or MeOH:H_2O (50:50 v/v)	2.5 mmol/L $NH_4CH_3CO_2$<br>100-200 mmol/L HCOOH	80-90 ppm	Andrianova (2018)[18]
Eugenol	Eugenol in IPA:diesel (9:1 v/v) with an internal standard (2-methoxy-4-methoxyphenol)	ESI-	AcN:H_2O (95:5 v/v)	100 mg/L NaOH	50-2500 ng/mL	Boes (2018)[19]
β-O-4′ model lignin compounds	β-O-4′ dimers were synthesized, and a stock solution (5 mM) was prepared for each dimer in AcN	ESI+	AcN; MeOH; IPA; n-propanol	0.5-10 mM LiCl (+ DMF or DMSO)	0.01-1 mM	Asare (2019)[20]
Enyzmatically processed Kraft lignin	Kraft lignin dissolved in 1:1 MeOH:H_2O	ESI-	MeOH:H_2O (1:1)	None	100–200 µg/mL	Echavarri-Bravo (2019)[21]
French pine lignin	0.1 mg pine lignin powder dissolved in 1 mL $CHCl_3$:MeOH (2:1)	APPI+	$CHCl_3$:MeOH (2:1)	None	~0.1 mg/mL	Mikhael (2020)[22]
Electrochemical lignin breakdown products	Analytes were dissolved in H_2O:MeOH (50:50 v/v)	ESI+<br>ESI-<br>APCI-<br>APPI-	H_2O: MeOH (50:50 v/v)	0.1% w/v NH_4HCO_2<br>None	N/A	Qi (2020)[23]
Dimeric lignin model compounds w/ β-O-4 linkages	Each compound was dissolved in MeOH at 1 mM	ESI-	MeOH	None	1 mM	Zhang (2020)[24]
Monomeric and dimeric lignin model compounds	Acetone:H_2O, 1,4-dioxane:H_2O, THF:H_2O (9:1, w/w)	APPI+<br>APPI-	Acetone:H_2O, 1,4-dioxane:H_2O, THF:H_2O (all 9:1, w/w)	Acetone, 1,4-dioxane, THF		Kosyakov (2021)[25]
Lignin model compounds with α-O-4 linkages	Stock solutions were prepared for all analytes with 1 mM final conc. in MeOH	ESI-<br>ESI+	MeOH	None<br>0.075% LiCl	0.075 mM	Sheng (2021)[26]

Qualitative visual evaluation of lignin samples

During sample preparation, observations were noted at all stages. A particularly key step in the process was observing how each powdered lignin sample interacted with each solvent mixture upon contact and mixing. Here, we wanted to make notes on apparent solubility, color, cloudiness (or "milkiness") and if there was any leftover residue. A visual inspection of all solvent mixtures for each lignin sample according to these categories allowed us to make a qualitative "variation score", where samples were ranked according to how much variation they exhibited after their initial mixing with the 8 different solvent mixtures: 1 = very little variation, 2 = some variation, 3 = dramatic variation between solvent mixtures. Variation was observed for 3 categories: Color, residue, and milkiness. We then summed these variation scores to give a total score out of 9, and then ranked the samples according to this total score. Table S2 displays the results.

Table S2: Qualitative visual evaluation of lignin samples – variation score 1-3

SAMPLE	COLOR	RESIDUE	MILKINESS	TOTAL SCORE (/9)
**LP3**	3	2	3	8
**LP9**	2	2	1	5
**LP5**	1	1	1	3
**LP7**	1	1	1	3

Comparison of results for LP3 across all solvent mixtures and ionization modes

For one sample, LP3 (hardwood lignin from organosolv process, no enzymatic degradation), we measured a full scan mass spectrum from *m/z* 100-2000 for all solvent mixtures A-J in both ESI- and ESI+ modes (and in ESI+ mode, with and without formic acid). From each spectrum, we subtracted the solvent blank spectrum, and exported a mass list to a text file. The total number of peaks observed in each file (corresponding to the number of non-background ions observed in each full scan mass spectrum) was used as a rough measure to ascertain how much ionizable lignin material each solvent mixture had extracted during sample preparation, and assisted in ionizing in the API source. For each sample set (ESI-, ESI+, and ESI+ with formic acid), the data was sorted according to the number of peaks observed in each spectrum, and each solvent mixture was given a ranking from 1-10 (with 10 corresponding to the mixture producing the greatest number of peaks, and 1 corresponding to the mixture producing the least). These rankings were summed from each sample set to produce an overall solvent ranking out of 30, and then sorted from highest to lowest rank to produce Table S3.

Table S3: Solvent rankings based on number of ions in spectra for negative mode and positive mode with and without formic acid for the LP3 sample

Code	Solvent mixture	Organic/Aqueous Ratio	Ranking
**D**	AcN:H_2O (1:1)	0.5	28
**B**	MeOH:H_2O (1:1)	0.5	25
**E**	MeOH:H_2O (3:1)	0.75	22
**C**	EtOH:H_2O (1:1)	0.5	20
**A**	Acetone:H_2O (1:1)	0.5	17
**J**	Acetone:MeOH:H_2O (1:1:2)	0.5	16
**G**	MeOH:H_2O (1:3)	0.25	14
**I**	Acetone:AcN:H_2O (1:1:2)	0.5	10
**H**	AcN:H_2O (1:3)	0.25	9
**F**	AcN:H_2O (3:1)	0.75	4

References

[1] D. V. Evtuguin, P. Domingues, F. L. Amado, C. P. Neto, A. J. F. Correia, **1999**, *53*, 525–528.
[2] D. V. Evtuguin, F. M. L. Amado, *Macromol. Biosci.* **2003**, *3*, 339–343.
[3] H. Önnerud, M. Palmblad, G. Gellerstedt, *Holzforschung* **2003**, *57*, 37–43.
[4] J. H. Banoub, M. Delmas, *J. Mass Spectrom.* **2003**, *38*, 900–903.
[5] J. H. Banoub, B. Benjelloun-Mlayah, F. Ziarelli, N. Joly, M. Delmas, *Rapid Commun. Mass Spectrom.* **2007**, *21*, 2867–2888.
[6] K. Morreel, H. Kim, F. C. Lu, O. Dima, T. Akiyama, R. Vanholme, C. Niculaes, G. Goeminne, D. Inze, E. Messens, J. Ralph, W. Boerjan, *Anal. Chem.* **2010**, *82*, 8095–8105.
[7] K. Morreel, O. Dima, H. Kim, F. C. Lu, C. Niculaes, R. Vanholme, R. Dauwe, G. Goeminne, D. Inze, E. Messens, J. Ralph, W. Boerjan, *Plant Physiol.* **2010**, *153*, 1464–1478.
[8] L. M. Amundson, R. J. Eismin, J. N. Reece, M. Fu, S. C. Habicht, A. B. Mossman, R. C. Shea, H. I. Kenttämaa, *Energy Fuels* **2011**, *25*, 3212–3222.
[9] M. Dauria, L. Emanuele, R. Racioppi, *Nat. Prod. Res.* **2012**, *26*, 1368–1374.
[10] L. J. Haupert, B. C. Owen, C. L. Marcum, T. M. Jarrell, C. J. Pulliam, L. M. Amundson, P. Narra, M. S. Aqueel, T. H. Parsell, M. M. Abu-Omar, H. I. Kenttämaa, *Fuel* **2012**, *95*, 634–641.
[11] E. Alsbou, B. Helleur, *Energy Fuels* **2014**, *28*, 578–590.
[12] D. S. Kosyakov, N. V. Ul'yanovskii, E. A. Anikeenko, N. S. Gorbova, *Rapid Commun. Mass Spectrom.* **2016**, *30*, 2099–2108.
[13] C. L. Marcum, T. M. Jarrell, H. Zhu, B. C. Owen, L. J. Haupert, M. Easton, O. Hosseinaei, J. Bozell, J. J. Nash, H. I. Kenttämaa, *ChemSusChem* **2016**, *9*, 3513–3526.
[14] T. K. F. Dier, K. Egele, V. Fossog, R. Hempelmann, D. A. Volmer, *Anal. Chem.* **2016**, *88*, 1328–1335.
[15] Y. L. Qi, R. Hempelmann, D. A. Volmer, *Anal. Bioanal. Chem.* **2016**, *408*, 4835–4843.
[16] D. S. Kosyakov, E. V. Ipatova, S. M. Krutov, N. V. Ul'yanovskii, I. I. Pikovskoi, *J. Anal. Chem.* **2017**, *72*, 1396–1403.
[17] H. Sheng, W. Tang, J. Gao, J. S. Riedeman, G. Li, T. M. Jarrell, M. R. Hurt, L. Yang, P. Murria, X. Ma, J. J. Nash, H. I. Kenttämaa, *Anal. Chem.* **2017**, *89*, 13089–13096.
[18] A. A. Andrianova, T. DiProspero, C. Geib, I. P. Smoliakova, E. I. Kozliak, A. Kubátová, *J. Am. Soc. Mass Spectrom.* **2018**, *29*, 1044–1059.
[19] K. S. Boes, M. S. Roberts, N. R. Vinueza, *J. Am. Soc. Mass Spectrom.* **2018**, *29*, 535–542.
[20] S. O. Asare, B. C. Lynn, *J. Mass Spectrom.* **2019**, *54*, 540–548.
[21] V. Echavarri-Bravo, M. Tinzl, W. Kew, F. Cruickshank, C. Logan Mackay, D. J. Clarke, L. E. Horsfall, *New Biotechnol.* **2019**, *52*, 1–8.
[22] A. Mikhael, T. D. Fridgen, M. Delmas, J. Banoub, *Rapid Communications in Mass Spectrometry* **2020**, *34*, e8910.
[23] Y. Qi, P. Fu, S. Li, C. Ma, C. Liu, D. A. Volmer, *Science of the Total Environment* **2020**, *713*, 136573.
[24] J. Zhang, E. Feng, W. Li, H. Sheng, J. R. Milton, L. F. Easterling, J. J. Nash, H. I. Kenttämaa, *Anal. Chem.* **2020**, *92*, 11895–11903.
[25] D. S. Kosyakov, I. I. Pikovskoi, N. V. Ul'yanovskii, *Analytica Chimica Acta* **2021**, *1179*, 338836.
[26] H. Sheng, W. Tang, J. Gao, J. Riedeman, M. Hurt, L. Yang, H. I. Kenttämaa, *Rapid Communications in Mass Spectrometry* **2021**, *35*, e9057.
